# In Situ Synthesis of β-Na_1_._5_Y_1_._5_F_6_: Er^3+^ Crystals in Oxyfluoride Silicate Glass for Temperature Sensors and Their Spectral Conversion and Optical Thermometry Analysis

**DOI:** 10.3390/molecules26226901

**Published:** 2021-11-16

**Authors:** Rajesh Dagupati, Robert Klement, Ramaraghavulu Rajavaram, José J. Velázquez, Dušan Galusek

**Affiliations:** 1 FunGlass, Alexander Dubček University of Trenčín, Študentská 2, SK-911 50 Trenčín, Slovakia; jose.velazquez@tnuni.sk (J.J.V.); dusan.galusek@tnuni.sk (D.G.); 2 Department of Physics, Annamacharya Institute of Technology & Sciences, Rajampet A.P-516115, India; ramaraghavulu@gmail.com; 3 Joint Glass Centre of the IIC SAS, TnUAD and FChPT STU, Trenčín, Študentská 2, SK-911 50 Trenčín, Slovakia

**Keywords:** glass, glass-ceramic, optical thermometry, up-conversion, down-conversion

## Abstract

Transparent oxyfluoride glass-ceramics (GCs) with embedded β-Na_1_._5_Y_1_._5_F_6_ crystals doped with Er^3+^ ions were fabricated by a melt-quenching method with subsequent heat-treatment. The structural characterizations and spectroscopic techniques were performed to verify the precipitation of β-Na_1_._5_Y_1_._5_F_6_ crystals and partition of the Er^3+^ dopant into the crystals. Bright green up-conversion (UC) emission was achieved in Er^3+^-doped glass-ceramic (Er-GC). Furthermore, the temperature-dependent visible UC behavior based on thermally coupled energy levels (TCLs) and non-thermally coupled energy levels (NTCLs) was also examined in the temperature range 298 k to 823 K with maximum relative sensitivity (*S_r_*) of 1.1% K^−1^ at 298 K for TCLs in Er-G and Er-GC samples.

## 1. Introduction

Temperature (T) is a fundamental parameter that must be measured precisely in many scientific and engineering fields [1,2,3]. Traditional temperature measuring devices, such as contact temperature sensors based on electrical changes in materials, have limitations in a variety of situations, such as nano- and micro-scale regimes, fast moving objects, corrosive environments, and inside cells, [4,5,6]. It is, therefore, essential to develop low-cost, portable, dependable, and safe temperature sensors for a wide range of applications. 

Among the various temperature measurement methods, which include the use of thermocouples, thermistors, and temperature resistance detectors (RTDs), the optical thermometry based on fluorescence intensity ratio (FIR) has received a lot of attention in the last decades because of its quick response, electromagnetic passivity, self-reference, and high sensitivity [4,7,8,9]. It also reduces the impact of the experimental conditions, such as fluorescence loss and fluctuation of the pumping power [10,11]. Kusama et al. [12] proposed FIR for the first time in 1976, and Shinn et al. [13] later provided the theoretical expression to its measurement in 1983. This technique advanced significantly in the twenty-first century [14,15,16]. 

The intensity ratio of two emission bands from thermally coupled levels (TCLs) of rare earth (RE^3+^) ions have been used to develop the FIR technique: it has been improved gradually in the past few decades. However, some inherent limitations of this method cannot be overcome [17,18]. Firstly, due to the limitation of rapid electrons exchange between the two monitoring energy levels (TCLs), the energy gap difference (ΔE) between them should be set at 200–2000 cm^−1^. This restriction in the energy gap can determine the thermometer’s upper limit of relative sensitivity (*S_r_* = ΔE/k_B_T^2^), which may be 1% or even lower [19,20]. Second, a narrow ΔE between TCLs would result in spectral overlap of the two monitoring peaks, which would be detrimental to signal recognition. These inherent limitations are unfavorable for further improvement of thermometer sensitivity [21]. A new strategy has been therefore proposed, based on the intensity ratio of non-coupled thermally (NTCLs) energy levels in the same and/or different luminescent centers to overcome the disadvantages of the TCLs route [22,23,24]. 

Among RE^3+^ ions, praseodymium [25], neodymium [3,9,16], europium [26], dysprosium [5], holmium [27], and erbium [11,20,22,24] have been used for optical temperature sensing due to the presence of thermally coupled levels. Among them, erbium (Er^3+^) is the most studied ion in glasses and glass-ceramics for optical thermometry due to its up-conversion (UC) fluorescence emissions upon excitation by infrared (IR) radiation at 980 nm. This IR excitation source shows some advantages with respect to ultraviolet (UV) excitation sources, i.e., it is commercially less expensive, more portable, and more suitable for biological applications. On the other hand, the efficiency of this system significantly depends on the environment in which the RE^3+^ ions are located, i.e., either in the regular crystal lattice or in disordered structure of glass with distorted coordination polyhedra around RE^3+^. In this regard, the radiative probabilities of emitting levels within RE^3+^ ions embedded in a crystalline phase of glass-ceramics, especially with low phonon energy (e.g., fluorides), are higher compared to glasses. Moreover, the advantage of glass-ceramics is that combines the properties of both glass and ceramics. Due to the promising luminescent properties of fluoride crystalline phases, RE^3+^-doped fluorides in glass-ceramics have attracted a lot of attention [28,29]. Among them, sodium yttrium fluoride phase in glass-ceramic became one of the most efficient host materials for UC among investigated fluoride glass-ceramics, with the incorporation of Er^3+^ for green emission [30,31].

Li et al. [32] studied up-conversion emission in transparent GCs containing β-NaYF_4_:Yb^3+^, Er^3+^ nanocrystals for optical thermometry in the range of 300–773 K. At 300 K, it has a maximum relative sensitivity of about 1.24% K^−1^. León-Luis et al. [11] studied the role of the host matrix, oxyfluoride glass and a fluoride-type nanocrystalline (α-NaYF_4_) GCs, on the thermal sensitivity of Er^3+^ luminescence in the temperature range from room temperature to 720 K. The best result was obtained with the glass, which produced a sensitivity of about 66 × 10^−4^ K^−1^ at 570 K due to the presence of highly distorted local symmetry environments of Er^3+^ ions and strong crystal-field strengths. However, to the best of our knowledge, there is no report on the synthesis of β-Na_1_._5_Y_1_._5_F_6_ crystals in 65.5SiO_2_-5Al_2_O_3_-3Y_2_O_3_-4Na_2_O-22NaF-0.5ErF_3_ glass with similar composition and optical thermometry analysis from thermally and non-thermally coupled energy levels for temperature sensing.

### 1.1. Fundamental Principle of FIR Sensing Thermometry Based on TCLs and NTCLs of Er^3+^ Ion

#### 1.1.1. Thermally Coupled Levels (TCLs)

**Figure 1:**
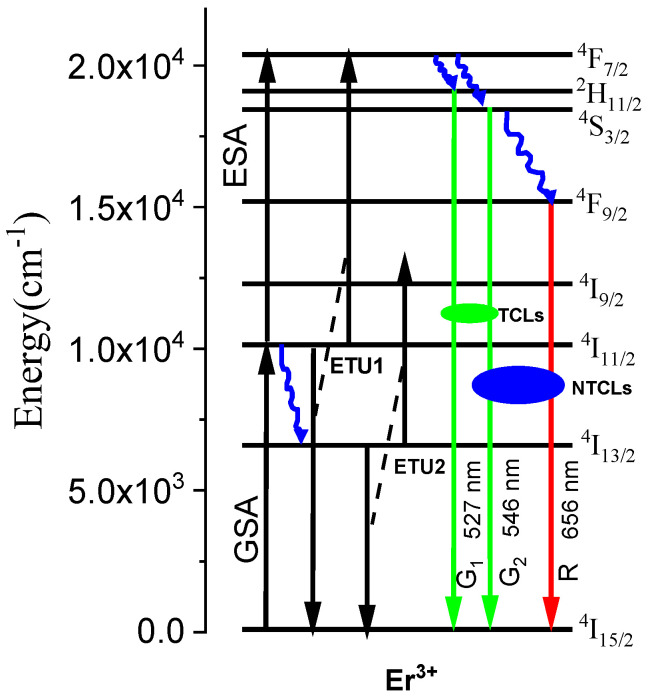
Possible energy transfer (ET) UC procedure in Er^3+^ ion under 980 nm laser excitation.

As illustrated in the Figure 1, under 980 nm laser excitation, two processes such as excited state absorption (ESA) and energy transfer UC (ETU1) are involved in green luminescence. In the ESA process, the electrons in ^4^I_15/2_ ground state of Er^3+^ are promoted to ^4^F_7/2_ excited state via successive two-step absorption process, i.e., (1) ground state absorption (GSA), and (2) excited state absorption (ESA) [13]. However, in ETU1 process, one of the Er^3+^ ions previously excited to ^4^I_11/2_ deexcited to the ground state, ^4^I_15/2_, while another ion was promoted to the ^4^F_7/2_ state because of mutual interaction. Finally, nonradiative transition from ^4^F_7/2_ state populates ^2^H_11/2_, ^4^S_3/2_ states. 

The most common mechanism for red UC is a nonradiative transition from the ^2^H_11/2_ and ^4^S_3/2_ states to the ^4^F_9/2_ state. Although NaYF_4_ phonon energy (360 cm^−1^) is taken into account, the energy gap between ^4^S_3/2_ and ^4^F_9/2_ states must be bridged by 7–8 phonons for this transition to be efficient [14]. Because of this, it is unlikely to have an impact on population of ^4^F_9/2_. On the other hand, GSA-populated Er^3+^ ions in the ^4^I_11/2_ state relax to the ^4^I_13/2_ state first and then populate the ^4^F_9/2_ emitting state through ETU2, as shown in Figure 1, producing red UC emission. The ETU2 is most likely a dominant mechanism in the present case. 

When the system is in a state of thermal equilibrium, electron populations in ^2^H_11/2_ and ^4^S_3/2_ two thermally coupled states follow Boltzmann distribution law, and the corresponding FIR can be expressed by the following equation [13]:

(1) $$\mathrm{FIR}=\frac{I_{524}}{I_{546}}=\frac{g_{H}\sigma_{H}\omega_{H}}{g_{S}\sigma_{S}\omega_{S}}\exp\left(\frac{-\Delta E}{k_{B}T}\right)=C\;\exp\left[\frac{-\Delta E}{k_{B}T}\right],\;$$

where I_524_ and I_546_ are integrated intensities corresponding to fluorescence transitions from the ^2^H_11/2_ and ^4^S_3/2_ levels to the ^4^I_15/2_ level, of Er^3+^ ion, respectively; g, σ, and ω, are the degeneracy, the emission cross-section, and the angular frequency of each level, respectively; C is a constant; ΔE is the energy gap between the ^2^H_11/2_ and ^4^S_3/2_ levels; k_B_ is Boltzmann constant, and T is absolute temperature. 

The energy gap ΔE between the ^2^H_11/2_ and ^4^S_3/2_ levels and the pre-exponential constant C can usually be calculated using one of two approaches: (1) intensity ratio of ^2^H_11/2_ and ^4^S_3/2_ levels (FIR) can be plotted against absolute temperature (T) and fitted by exponential function; and (2) the linear fit of the graph plotted ln (FIR) vs. 1/T. The ΔE values obtained from these two approaches identical [33,34]. The first approach has been adapted in the present case. The ΔE and C parameters are vital factors for the absolute sensor sensitivity (*S_a_*) as well as the relative sensitivity (*S_r_*) of temperature detection, as defined by the following equations [11]:

(2) $$S_{a}=\frac{d\left(\mathrm{FIR}\right)}{\mathrm{dT}}=\mathrm{FIR}\left(\frac{\Delta E}{k_{B}T^{2}}\right),\;$$

(3) $$S_{r}=\frac{1}{\mathrm{FIR}}\frac{d\left(\mathrm{FIR}\right)}{\mathrm{dT}}=\frac{\Delta E}{k_{B}T^{2}}.$$

#### 1.1.2. Non-Thermally Coupled Levels (NTCLs)

The FIR sensing thermometry is based on NTCLs, which involve two independent luminescent excited levels, in the present case ^4^S_3/2_ and ^4^F_9/2_ levels in Er^3+^ ion, each with its own photoluminescence temperature dependence. Because these two luminescent excited levels are electronically independent in terms of kinetics, there is no energy transfer (ET) between them on the time scale of their luminescence (1/τ ≫ k_ET_) [16]. 

To further demonstrate the temperature sensing mechanism of NTCLs by FIR temperature sensing method, the temperature properties of ^4^S_3/2_ and ^4^F_9/2_ levels are analysed using the polynomial function below.

(4) $$\mathrm{FIR}=\frac{I_{658}}{I_{546}}=A+B\times T+{C\times T}^{2},$$

where, A, B, and C are the fitting parameters of polynomial. The sensitivities *S_a_* and *S_r_* based on NTLC could then be calculated by taking the first derivative of the FIR function (Equation (4)) and Equations (2) and (3). 

In the current study, we used melt quenching followed by controlled heat treatment to create β-Na_1_._5_Y_1_._45_F_6_ Er_0_._05_ crystals in oxyfluoride silicate glasses. The dynamics of excited states of Er^3+^ ions, as well as high-temperature sensing properties, have been investigated in a temperature range of 298–823 K using TCLs and NTCLs energy levels transition of Er^3+^ ions in glass and GCs based on FIR technique. The phase composition of the as prepared GC has been examined using Rietveld refinement of diffraction patterns obtained from X-ray powder diffraction (XRD) measurements. The thermal stability of both species has been investigated using differential scanning calorimetry (DSC). Er^3+^-doped oxyfluoride silicate GC has been shown to have excellent spectral/thermal stability and high-temperature sensing performance in the investigated temperature range. 

## 2. Results and Discussion

### 2.1. Thermal and Structural Characterization 

**Figure 2:**
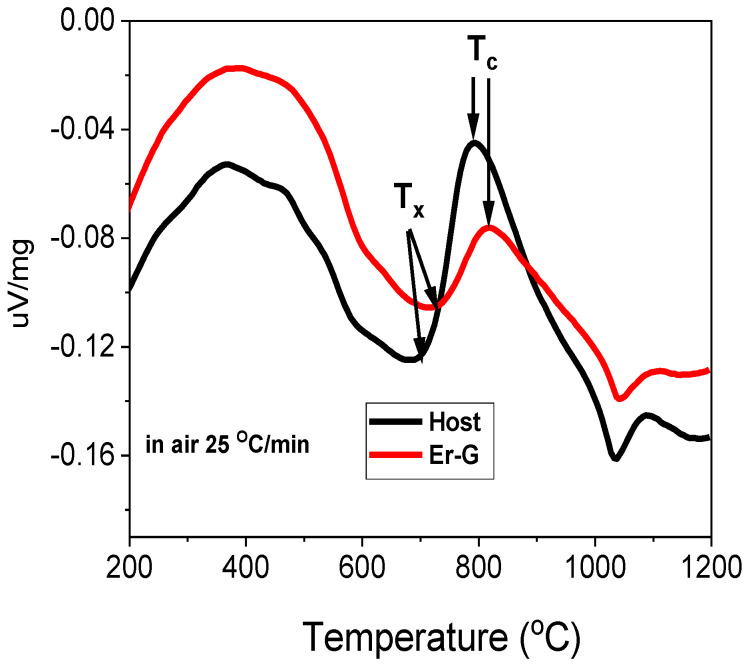
DTA curves of host and Er-doped oxyfluoride silicate glass (Er-G).

Figure 2 depicts the DTA curves of the host and Er-G. Based on the profiles, the onset crystallization temperature (T_x_) is around 695 °C and 728 °C and the bulk crystallization temperature (T_c_) is around 795 °C and 820 °C for host and Er-G, respectively. The glass transition temperature (T_g_) could not be discerned from the DSC curves. Therefore, dilatometry analysis for both host and Er-G was carried out at a heating rate of 5 °C/min (not shown) and T_g_ was determined to be identical (~510 °C) for both compositions. 

**Figure 3:**
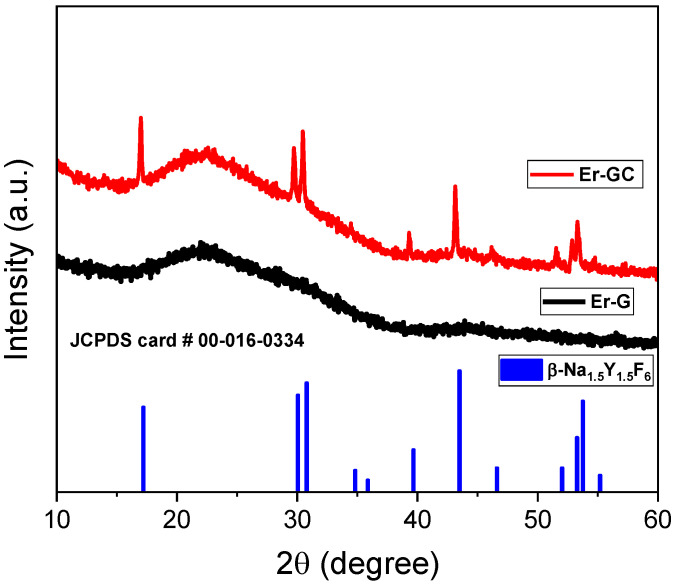
X-ray diffraction patterns of Er-doped oxyfluoride silicate glass (Er-G) and glass-ceramic (Er-GC) heat-treated at 650 °C for 40 h.

**Figure 4:**
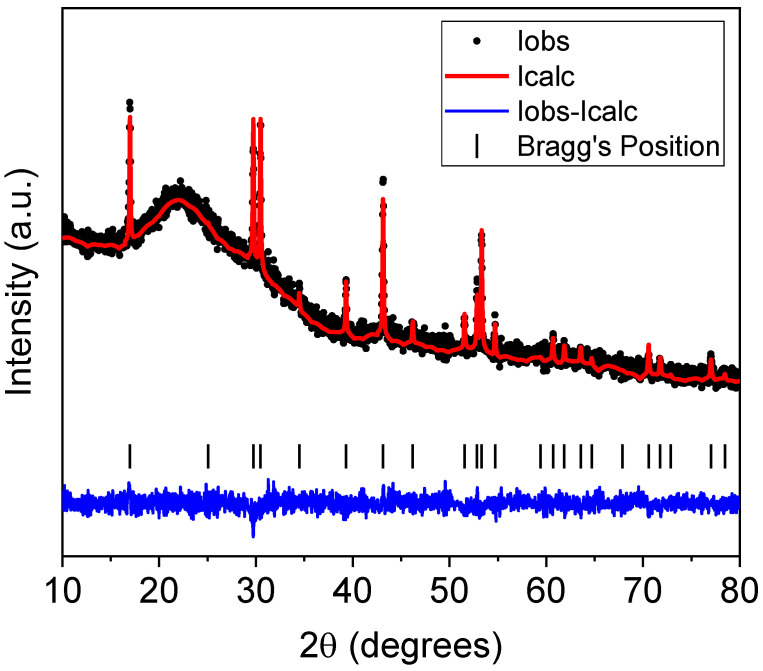
Rietveld-refined XRD spectrum of Er-GC (heat-treated at 650 °C for 40 h).

The XRD patterns of Er-G and Er-GC after the heat treatment (40 h) are shown in Figure 3. The Er-G diffraction pattern showed only a broad halo, indicating its non-crystalline nature. When Er-G was heat-treated for 40 h at 650 °C, diffraction maxima were observed, that were in a good agreement with the JCPDS card # 00-016-0334, indicating the presence of a pure hexagonal sodium yttrium fluoride phase in the GCs. Based on the XRD data, the Rietveld method was used to refine the crystal structure of the Er-GC materials, as shown in Figure 4. The results are presented in Table 1. 

The Figure 4 shows that the fitting has good convergence, as indicated by the low R factors and a close match between the calculated and experimental patterns. The Rietveld refinement confirmed that the Er-GC contains hexagonal sodium yttrium rare-earth fluoride phase, Na_1_._5_Y_1_._45_Er_0_._05_F_6_. The data are in good agreement with the works of Krämer et al. [35] and Grzechnik et al. [36] who thoroughly investigated the crystal structure of Na_1_._5_Y_1_._5_F_6_. No traces of cubic phase or other impurity phase was detected.

### 2.2. Optical Characterization

**Figure 5:**
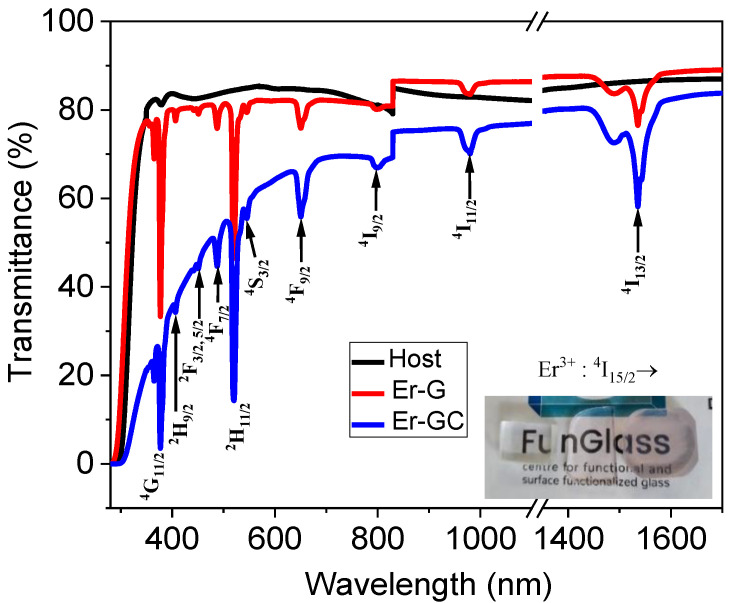
UV–vis–NIR transmittance spectra of the host, Er-G and Er-GC.

The transmittance spectra of the host, Er-G, and Er-GC samples in the wavelength range from 280 to 1700 nm are shown in Figure 5. Er^3+^ transmission bands at 1537, 980, 799, 650, 545, 522, 489, 451, 406, and 375 nm were assigned to transitions from the ground state, ^4^I_15/2_, to excited states, ^4^I_13/2_, ^4^I_11/2_, ^4^I_9/2_, ^4^F_9/2_, ^4^S_3/2_, ^2^H_11/2_, ^4^F_7/2_, ^2^F_5/2, 3/2_, ^2^H_9/2_, and ^4^G_11/2_, respectively. No change in band position has been observed, but the transmittance of Er-GC decreased in comparison to host glass. The daylight picture of the host, Er-G, and Er-GC are shown in the bottom inset in Figure 5 (from left to right).

**Figure 6:**
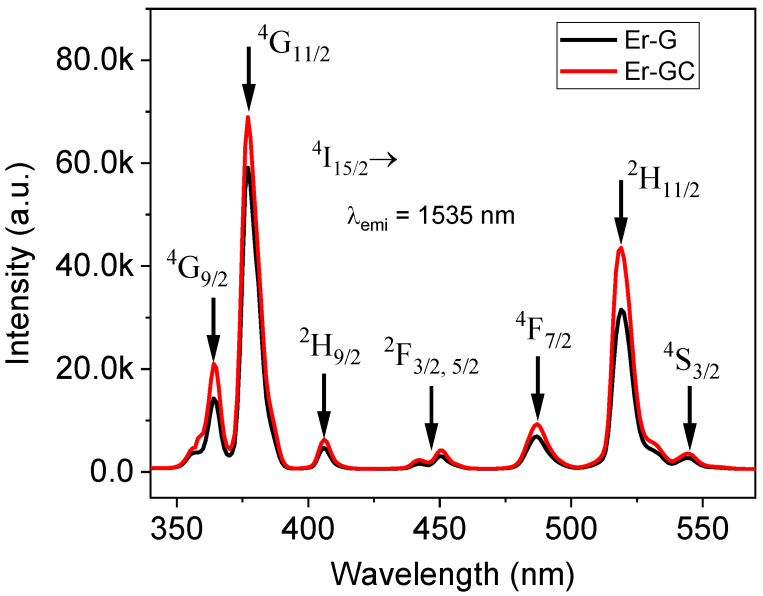
Photoluminescence excitation spectra (PLE) of Er-G and Er-GC measured under emission wavelength 1535 nm.

The photoluminescence excitation spectra (PLE) in the visible region and near-infrared region (NIR) for Er-G and Er-GC were measured at the emission wavelength of 1535 nm, as shown in Figure 6. The intensity of all excitation bands in glass-ceramic (Er-GC) increased when compared to that of the glass due to a change of local environment of the Er^3+^ ions during crystal precipitation. The band at 375 nm (^4^I_15/2_→^4^G_11/2_) was selected from among all excitation bands to record the emission spectra. 

**Figure 7:**
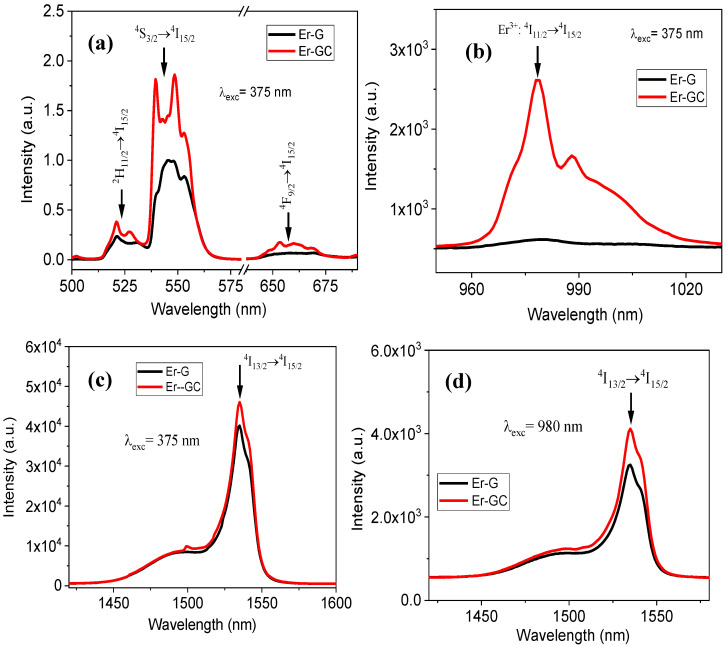
Photoluminescence spectra (PL) in (**a**) visible, (**b**,**c**) NIR region measured under excitation wavelength 375 nm and (**d**) NIR region measured under excitation wavelength 980 nm for Er-G and Er-GC.

Figure 7 depicts photoluminescence (PL) spectra recorded at the excitation wavelength 375 nm for the visible and near-infrared (NIR) spectral regions and at the excitation wavelength 980 nm for the NIR region. The intensities of the emission bands in glass-ceramic (Er-GC) are higher than those observed in the glass (Er-G). Furthermore, crystal filed splitting in Stark components has been observed in Er-GC at emission transitions, ^4^S_3/2_→^4^I_15/2_ and ^4^I_11/2_→^4^I_15/2_. The intensity and crystal field splitting in emission transitions changed as a result of the phase transition induced by thermal treatment, which changed the immediate environment of the Er^3+^ ions from an oxyfluoride environment in Er-G to a pure fluoride environment in Er-GC, and serves as an indicator of incorporation of the Er^3+^ ions into the NCs [11,28]. 

**Figure 8:**
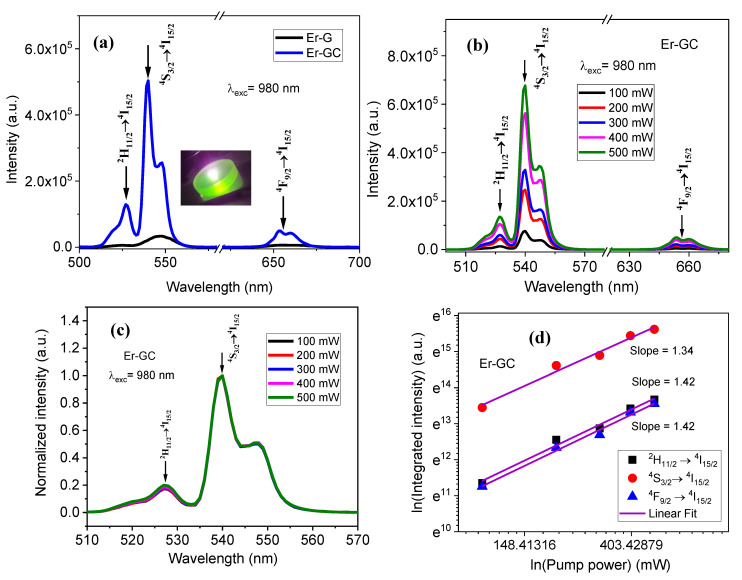
Up-conversion (UC) PL spectra (**a**) at 500 mW pump power for Er-G and Er-GC, and inset is picture of Er-GC exited with laser at 980 nm (**b**) at different pump power for Er-GC, (**c**) normalized at different pump power for Er-GC, (**d**) ln (UC intensity) v.s. ln (pump power) for Er-GC measured under 980 nm excitation wavelength.

The UC emission spectra of Er-G and Er-GC upon 980 nm laser diode excitation with a fixed laser power of 500 mW (8.33 W/cm^2^) in the wavelength region between 500 and 700 nm are shown in Figure 8a. The picture of the Er-GC excited with a 980 nm laser diode is shown as inset in Figure 8a and observed by naked eye as strong green. There are three UC emission bands in the visible region centered at 527, 546, and 656 nm, corresponding the ^2^H_11/2_→^4^I_15/2_, ^4^S_3/2_→^4^I_15/2_ and ^4^F_9/2_ →^4^I_15/2_ transitions, respectively, which are labelled as G_1_, G_2_ and R, respectively, and are schematically represented in Figure 1. It is worth mentioning that the intensity of all UC emission bands is higher in Er-GC than in Er-G. This improvement is attributed to a decrease in the multi phonon relaxation rates of ^2^H_11/2_, ^4^S_3/2_, and ^4^F_9/2_ levels when environment of Er^3+^ ions was changed from an oxyfluoride environment in Er-G to a pure fluoride environment in Er-GC. Furthermore, each emission transition in Er-GC shows more than one component due to Stark splitting of Er^3+^:^2^H_11/2_, ^4^S_3/2_, and ^4^F_9/2_ levels [37]. 

The pump-power dependent UC emission spectra for Er-GC after 980 nm excitation, shown in Figure 8b, help to determine the number of pump photons responsible for various UC emission processes. It is worthy to mention that the emission spectra of Er-GC exhibited the same features for all laser powers ranging from 100 mW (1.67 W/cm^2^) to 500 mW (8.33 W/cm^2^), except for an increase in their intensities. This increase in intensity across all emission bands is due to an enrichment in the population of excited states at high power [38]. To investigate the heating effect caused by a 980 nm laser excitation source, we normalized the green up-conversion emission under different pump powers and examined the spectral profiles. The spectral profiles remain constant, as shown in Figure 8c. This is possible if the heating effect is negligible.

The following expression has been used to calculate the number of photons contributed during the UC process:

I = k (P)^n^, (5)

where ‘k’ is the constant and ‘n’ is the number of photon involved in the UC process. According to the above expression, the linear fit of the data points in the plot relating logarithmic values of pump powers ln (P) to corresponding UC emission intensity ln (I) of emission bands (Figure 8d) yields the slope value, which is equivalent to the number of photons involved in the up-conversion process. The slope values for green UC transitions, G_1_ and G_2_, are 1.42 and 1.34, respectively, whereas the slope for red UC emission transition, R, is 1.42. The slope value less than 2 is commonly observed at high pump power density, when the depopulation of the excited levels by up-conversion processes is comparable to the radiative decay rate. In these conditions the observed can be fitted by considering powers lower than n [39]. On the other hand, as discussed in Section 1.1.1, the states ^2^H_11/2_, ^4^S_3/2_, and ^4^F_9/2_ are populated not only by excited state absorption (ESA), but also by energy transfer up-conversion (ETU). As a result, the slopes are less than 2. The obtained slope values indicate that two photons are involved in the green and red up-conversion emission. 

**Figure 9:**
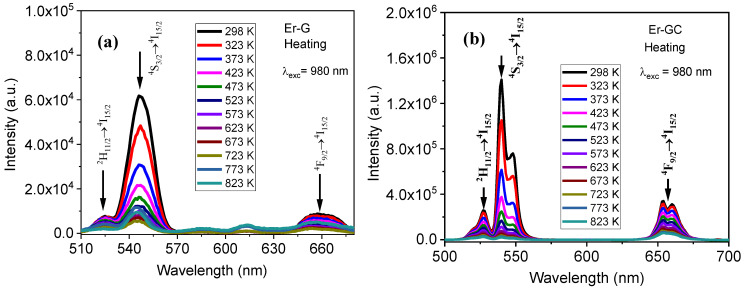
UC PL spectra measured at different temperatures when heating the samples, (**a**) Er-G and (**b**) Er-GC.

The UC emission spectra were recorded by heating and cooling the samples in the temperature range 298 K and 823 K upon excitation 980 nm to investigate the temperature sensing ability of Er-G and Er-GC samples based on FIR of TCLs and NTCLs. The results are shown in Figure 9. 

**Figure 10:**
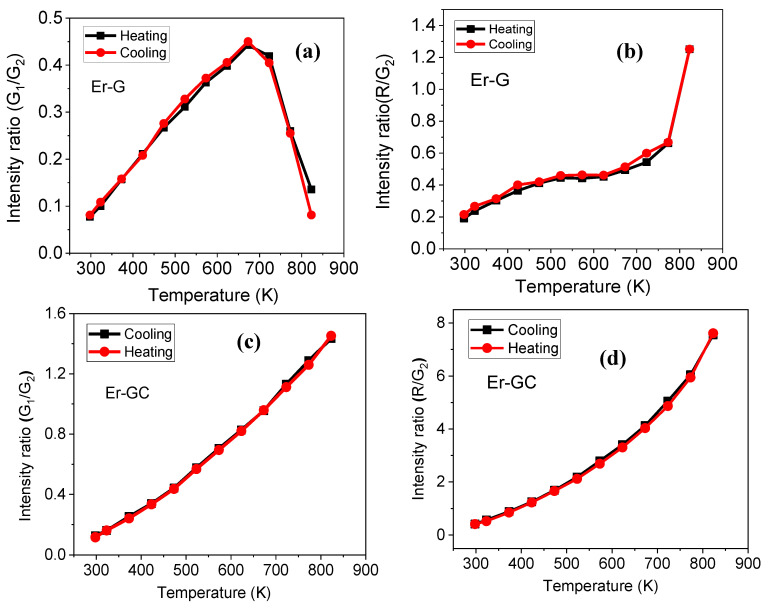
Intensity ratio of transitions (**a**,**c**) G_1_/G_2_ and (**b**,**d**) R/G_2_ in Er-G and Er-GC samples.

The FIR of transitions, G_1_/G_2_ (attributed from TCLs) increased with increasing temperature from 298 K to 673 K, then decreased in the Er-G sample (Figure 10a). On the other hand, the FIR of the same transitions in the Er-GC sample increased with increasing temperature (Figure 10c). In Er-G and Er-GC samples, FIR of transitions, R/G_2_ (attributed from NTCLs) increased (Figure 10b,d) with increasing temperature from 298 K to 823 K. Above 700 K, the FIR of transitions, G_1_/G_2_, and R/G_2_ rapidly changes in Er-G. This could be attributed to the fcat that the intensity ratio of G_1_/G_2_ lines no longer follows the Boltzmann distribution due to thermal saturation, and the kinetics of green and red emission may differ at high and low temperatures. Furthermore, when Er-G and Er-GC samples were cooled, the FIR of TCLs and NTCLs levels are the same as in heated samples (except at 823 K in Er-G).

**Figure 11:**
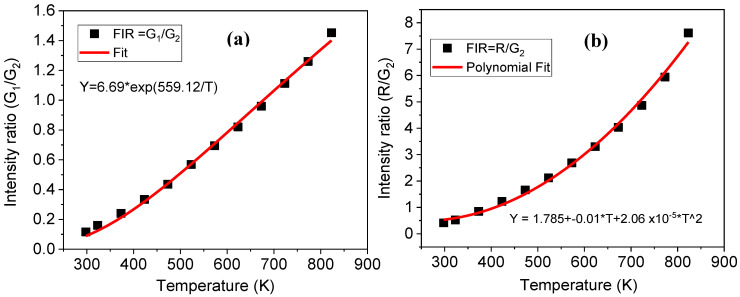
(**a**) Exponential fit of FIR of G_1_/G_2_, and (**b**) polynomial fit of FIR of R/G_2_ transitions against temperature for Er-GC sample.

The FIR of transitions attributed to TCLs (Figure 10a,b) and NTCLs (Figure 10b,d) against temperature (for Er-G sample taken into account up to 673 K) was fitted by exponential equation, Equation (1), and polynomial equation, Equation (4), respectively, shown in Figure 11 for Er-GC sample and in Figure S1 in Supplementary Material for the Er-G sample. 

**Figure 12:**
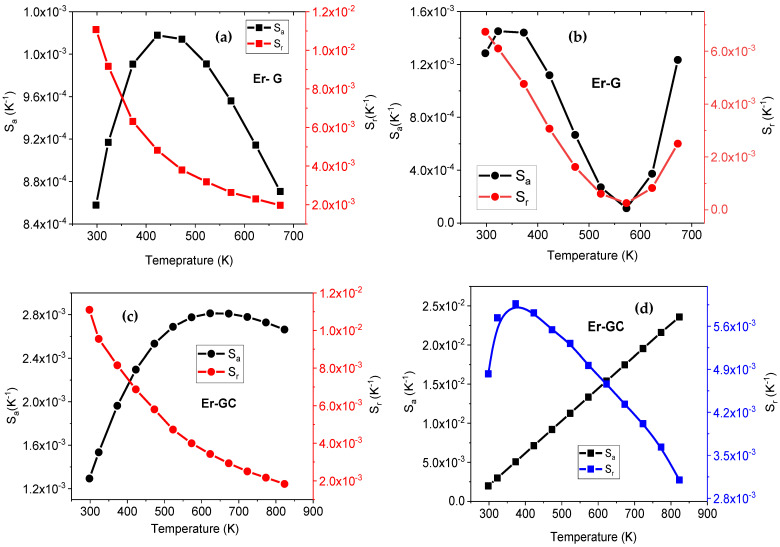
Absolute and relative temperature sensitivity versus temperature: (**a**,**c**) are TCLs of Er^3+^ in Er-G and Er-GC and (**b**,**d**) are NTCLs of Er^3+^ in Er-G and Er-GC, respectively.

The absolute (S_a_) and relative (S_r_) sensitivity of Er-G and Er-GC samples versus temperature were calculated using Equations (2) and (3). The results for Er-G and Er-GC are shown in Figure 12, respectively. In the Er-G sample, the S_a_ reached a maximum of about 0.00102 K^−^^1^ for TCLs at 423 K and 0.00145 K^−^^1^ for NTCLs at 323 K. In Er-GC sample, S_a_ has reached a maximum of approximately 0.00281 K^−^^1^ for TCLs K at 623 K and 0.02364 K^−^^1^ for NTCLs at 823 K. In contrast to S_a_, the S_r_ decreases with increasing temperature for TCLs and its maximum approximately equals (1.1% K^−^^1^) in Er-G and Er-GC at 300 K. However, the maximum S_r_ obtained from NTCLs of Er^3+^ ion is less than that obtained from TCLs in both Er-G and Er-GC, which is contrary to what was expected (>1.1% K^−^^1^). 

The maximum value of the absolute thermal sensitivity (S_a_), the relative thermal sensitivity (S_r_), the temperature range (ΔT), energy spacing (∆E), and the excitation wavelength (λ_exc_) for different glass and GC host matrices doped with Er^3+^ or co-doped with Er^3+^/Yb^3+^ are presented in Table 2. The maximum S_a_ value of Er-GC is in agreement with the results reported for Er^3+^/Yb^3+^: SiO_2_-BaF_2_-ZnF_2_ glass [40], and Er^3+^: PbO-Ga_2_O_3_-SiO_2_ glass [41]. However, it is lower than the results reported for Er^3+^/Yb^3+^: NaYb_2_F_7_—GC [42] and Er^3+^/Yb^3+^: NaLu_2_F_7_—GC [43] and higher than the results reported for Er^3+^/Yb^3+^: NaYF_4_—GC [44] and Er^3+^/Yb^3+^: fluoro phosphates glass [45]. 

## 3. Materials and Methods

### 3.1. Glass and Glass-Ceramic Preparation

A glass with the chemical composition 65.5SiO_2_-5Al_2_O_3_-3Y_2_O_3_-4Na_2_O-22NaF-0.5ErF_3_ (in mol%) was prepared by the conventional melting and subsequent quenching of the melt. 50 g batch of analytically pure (99.9% purity or higher) raw materials, SiO_2_, Al_2_O_3_, Y_2_O_3_, Na_2_O, NaF, and ErF_3_ were homogenized using rotating mill for 2 h. The mixed raw materials were calcined at 1200 °C for 2 h and, subsequently melted at 1680 °C for 2 h in a covered Pt-10% Rh crucible in a high-temperature elevator furnace in ambient atmosphere. The crucible was covered with a platinum lid to prevent fluorine volatilization. The melt was poured onto an iron plate and annealed at 450 °C for 2 h to release internal stress. The obtained glass slab was cut into several pieces of circular (diameter 0.3 cm) or rectangular shape. The bulk glass pieces were nucleated at 510 °C for 4 h and subsequently heat-treated at 650 °C for 40 h, using a heating rate of 10 °C/min to prepare the corresponding GCs. Finally, as prepared glass and GCs were polished with CeO_2_ paste for optical measurements. For better understanding the as prepared samples are labeled as follows:

Host: Undoped glass

Er-G: Er^3+^-doped glass

Er-GC: Er^3+^-doped glass-ceramic

### 3.2. Structural and Optical Characterization 

The X-ray diffraction (XRD) patterns of glass and GCs were recorded using an X-ray diffractometer (Panalytical Empyrean DY1098), operated at 40 kV and 45 mA with Cu Kα radiation (λ = 0.15405 nm) in steps of 0.02° in the 2θ angular range from 10° to 80°. The data was evaluated using the software High Score Plus with the crystallographic open database (COD 2018).

The differential thermal analysis (DTA) measurement for glass and GCs were performed using the Netzsch STA 449 F1 Jupiter simultaneous thermal analyzer in the temperature range between 35 and 1200 °C, at a heating rate of 25 °C min^−1^, in ambient atmosphere. 

The UV–Vis–NIR transmittance spectra of the synthesized glass and GCs were recorded using a Cary 5000 spectrometer (Agilent Technologies, USA) in the spectral range 200–1800 nm with the step of 0.5 nm. 

The photoluminescence spectra in visible (VIS) region at room temperature were recorded on the Fluorolog FL3-21 fluorescence spectrophotometer (Jobin-Yvon Horiba, France), in front face mode (backscattering geometry), with an Xe lamp (450 W) used as the excitation light source. The signals were collected using a photomultiplier tube R928 detector. 

The excitation pump power and temperature-dependent UC emission spectra were recorded using the same instrument, using a diode laser operating at 978 nm as the excitation source. To avoid the heating effect caused by the laser, the UC spectra were recorded in a short time interval. For high-temperature UC luminescence spectra measurement, a circular shape sample 0.3 cm diameter was set in a Linkam heating stage (TS1500, Linkam Scientific, UK; temperature range RT to 1500 °C, temperature precision < 1 °C) coupled with an integration sphere to collect the emitted light and connected to the fluorescence spectrometer via a glass fiber. 

## 4. Conclusions

In this work, hexagonal sodium yttrium fluoride crystalline phase in oxyfluoride silicate glass doped with Er^3+^ ion has been synthesized by melt quenching and subsequent heat treatment. According to Rietveld refinement of XRD spectra the main crystalline phase in Er-GC was β-Na_1_._5_Y_1_._45_Er_0_._05_F_6_. The temperature-dependent visible UC emission was studied over a wide temperature range of 298–823 K, with the maximum absolute thermal sensitivity (*S_a_*) of Er-G being 1.01 × 10^−^^3^ K^−^^1^ at 423 K for TCLs and 1.45 × 10^−^^3^ K^−^^1^ at 323 K for NTCLs. The *S_a_* has reached a maximum of 2.81 × 10^−^^3^ K^−^^1^ at 623 K for TCLs and 23.64 × 10^−^^3^ K^−^^1^ at 823 K for NTCLs in the Er-GC sample. The relative thermal sensitivity (*S_r_*) obtained from the TCLs of Er^3+^ ion is 1.1% K^−^^1^ (at 298 K) in Er-G and Er-GC. However, the maximum *S_r_* obtained from NTCLs in Er-G and Er-GC is lower than that obtained from TCLs. Based on these results we conclude that Er-GC is a promising candidate for up-conversion luminescence temperature sensors.

## Figures and Tables

**Table 1:** Crystal parameters of the hexagonal sodium ytrium fluoride crystals in Er-GC obtained by Rietveld analysis.

Parameter	Er-GC
Phase	Na_1_._5_Y_1_._45_Er_0_._05_F_6_
Space group	P-6 (174)
a (Å)	5.9831
b (Å)	5.9831
c (Å)	3.5350
α (degrees)	90
β (degrees)	90
γ (degrees)	120
V (Å^3^)	109.6094
R_e_ (%)	3.42695
R_p_ (%)	2.52878
R_wp_ (%)	3.23458

**Table 2:** Values of the maximum absolute sensitivity, *Sa*, and relative sensitivity, *Sr*, at starting temperature, energy spacing (∆E), experimental temperature ranges of different Er^3+^ and Er^3+^/Yb^3+^ co-doped materials using the fluorescence intensity ratio (FIR) method based on the green emissions of the Er^3+^ ion.

Sensing Materials	λ_exc (_nm)	TCLs			NTCLs		Experimental Temperature Range (K)	Ref
		∆E (cm^−1^)	Maximum of S_a_ (×10^−3^ K^−1^)	Maximum of S_r_ (% K^−1^)	Maximum of S_a_ (× 10^−3^ K^−1^)	Maximum of S_r_ (% K^−1^)		
Er^3+^:Na_1_._5_Y_1_._5_F_6_-GC	980	388	2.81 @ 623	1.1	23.64 @ 823	0.6	298–823	Present work
Er^3+^/Yb^3+^: SiO_2_-BaF_2_-ZnF_2_ glass	980	719	2.70 @ 513	1.2	---	---	291–450	Feng et al. [40]
Er^3+^: PbO-Ga_2_O_3_-SiO_2_ glass	980	---	2.64 @ 590	---	---	---	300–630	Pisarski et al. [41]
Er^3+^/Yb^3+^: NaYb_2_F_7_-GC	980	852	3.06 @ 600K	1.3	---	---	300–773	Hua et al. [42]
Er^3+^/Yb^3+^: NaLu_2_F_7_-GC	980	861	4.73 @ 620 K	1.3	---	---	300–773	Cao et al. [43]
Er^3+^/Yb^3+^: NaYF_4_-GC	980	774	2.30 @ 573	1.2	---	---	298–693	Jiang et al. [44]
Er^3+^/Yb^3+^:fluoro phosphate glass	980	390	1.5 @ 279	---	---	---	130–490	Lai et al. [45]

## Data Availability

Not applicable.

## Supplementary Materials

The following are available online, Figure S1: (**a**) Exponential fit of FIR of G_1_/G_2_, and (**b**) polynomial fit of FIR of R/G_2_ transitions against temperature for Er-G sample.

## Sample Availability

Samples are available from the authors on responsible request.
