# Supplementary material for: In Situ Synthesis of β-Na1.5Y1.5F6: Er3+ Crystals in Oxyfluoride Silicate Glass for Temperature Sensors and Their Spectral Conversion and Optical Thermometry Analysis
Source: Molecules. 2021 Nov 16;26(22):6901. doi: 10.3390/molecules26226901 (PMC8619172; doi:10.3390/molecules26226901)
Supplement: Supplementary file 1 [file molecules-26-06901-s001.zip › molecules-1443243-supplementary.pdf]

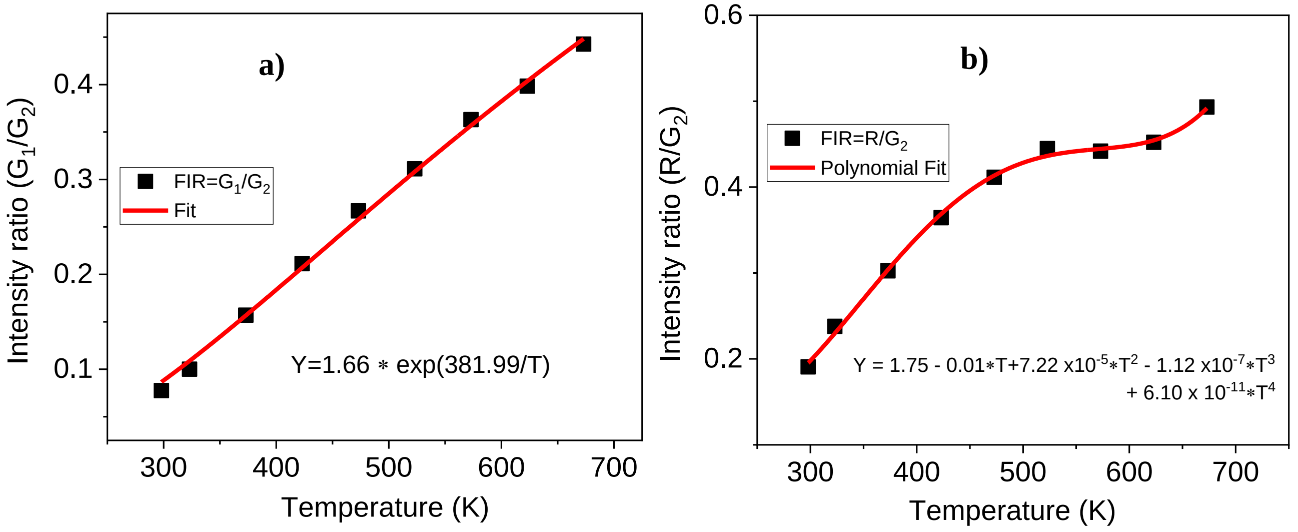

Figure S1: a) Exponential fit of FIR of G_1/G_2, and **b)** polynomial fit of FIR of R/G_2 transitions against temperature for Er-G sample.
